# Image-Guided Radiotherapy for Cardiac Sparing in Patients with Left-Sided Breast Cancer

**DOI:** 10.3389/fonc.2014.00257

**Published:** 2014-09-23

**Authors:** Claire Lemanski, Juliette Thariat, Federico L. Ampil, Satya Bose, Jacqueline Vock, Rick Davis, Alexander Chi, Suresh Dutta, William Woods, Anand Desai, Juan Godinez, Ulf Karlsson, Melissa Mills, Nam Phong Nguyen, Vincent Vinh-Hung, The International Geriatric Radiotherapy Group

**Affiliations:** ^1^Department of Radiation Oncology, Centre Val d’Aurelle, Montpellier, France; ^2^Department of Radiation Oncology, University of Nice, Nice, France; ^3^Department of Radiation Oncology, Louisiana State University, Shreveport, LA, USA; ^4^Department of Radiation Oncology, Howard University, Washington, DC, USA; ^5^Department of Radiation Oncology, Lindenhofspital, Bern, Switzerland; ^6^Michael D. Watchtel Cancer Center, Oshkosh, WI, USA; ^7^Department of Radiation Oncology, University of West Virginia, Morgantown, WV, USA; ^8^Department of Radiation Oncology, Medicine and Radiation Oncology PA, San Antonio, TX, USA; ^9^Department of Radiation Oncology, Richard A. Henson Institute, Salisbury, MD, USA; ^10^Department of Radiation Oncology, Akron City Hospital, Akron, OH, USA; ^11^Florida Radiation Oncology Group, Department of Radiation Oncology, Jacksonville, FL, USA; ^12^Department of Radiation Oncology, Marshfield Clinic, Marshfield, WI, USA; ^13^Department of Radiation Oncology, University of Arizona, Tucson, AZ, USA; ^14^Department of Radiation Oncology, University of Martinique Hospital, Martinique, France

**Keywords:** breast cancer, left breast, cardiac toxicity, IGRT

## Abstract

Patients with left-sided breast cancer are at risk of cardiac toxicity because of cardiac irradiation during radiotherapy with the conventional 3-dimensional conformal radiotherapy technique. In addition, many patients may receive chemotherapy prior to radiation, which may damage the myocardium and may increase the potential for late cardiac complications. New radiotherapy techniques such as intensity-modulated radiotherapy (IMRT) may decrease the risk of cardiac toxicity because of the steep dose gradient limiting the volume of the heart irradiated to a high dose. Image-guided radiotherapy (IGRT) is a new technique of IMRT delivery with daily imaging, which may further reduce excessive cardiac irradiation. Preliminary results of IGRT for cardiac sparing in patients with left-sided breast cancer are promising and need to be investigated in future prospective clinical studies.

## Cardiac Toxicity Following Left Breast Cancer Irradiation

Patients with left-sided breast cancer are at risk of long-term cardiac complications following irradiation. In a study of 2168 breast cancer patients who had post-operative breast irradiation, the risk of major coronary events was significantly higher among the patients who had radiation to the left breast. The rates of major coronary events were proportional to the mean heart dose and continued into the third decade after surgery (1). All study patients were treated with the conventional 3-dimensional conformal radiotherapy technique (3D-CRT), which has been reported to increase radiation dose to the heart despite physician attempts to shield the heart from the radiation (2). Indeed, a higher risk of cardiac mortality was observed among patients with left-sided breast cancer compared to the ones with right breast cancer following post-operative irradiation (3). Even though recent studies suggest that the mortality of left breast irradiation was not different than the right breast after 1993, long-term cardiac damage frequently occurs after two decades, prompting some caution that these adverse events may arise in the next decade as the patients become older (4). Significant reduction in myocardial function was observed in patients with left breast cancer following radiation dose as low as 3 Gy 2 months after treatment (5). Set up variation with two tangent fields has been demonstrated to expose the myocardium to a higher dose of radiation leading to myocardial hypoperfusion (6). Radiation-induced myocardial damage is often clinically silent even when there was significant reduction of the left ventricular ejection fraction (7). The damage to the heart may be worse in patients with pre-existing cardiovascular conditions such as high blood pressure or left ventricle dysfunction (8). In addition, in patients who required adjuvant chemotherapy in addition to radiation following breast cancer surgery, cardiac toxicity may be compounded from the radiosensitization effect of chemotherapy.

Cardiac toxicity following chemotherapy and radiotherapy for breast cancer occurs late and increases on long-term follow-up with serial echocardiography (9). As radiation damage to the myocardium is primarily due to the inflammation and scarring of the heart microvascular structure, which leads to hypoperfusion and ventricular dysfunction, minimizing irradiation of the normal heart without compromise of target coverage should be the primary objective in patients with left-sided breast cancer (10, 11). To achieve this goal, new radiotherapy techniques such as intensity-modulated radiotherapy (IMRT) and image-guided radiotherapy (IGRT) have been introduced to limit cardiac toxicity.

## Intensity-Modulated Radiotherapy for Cardiac Protection in Patients with Left-Sided Breast Cancer

The ability to modulate radiation beams during radiation treatment has been investigated extensively to spare normal organs from excessive radiation. Breast cancer IMRT is usually delivered using dynamic multileaf collimation (MLC) or as a limited number of static MLC segments delivered sequentially in the step-and-shoot fashion. The beam modulation allows for a homogeneous dose distribution within the breast avoiding areas of high dose leading to less side effects and possibly better cosmesis in randomized studies (12, 13). Compared to 3D-CRT, IMRT may significantly reduce radiation dose to the myocardium in patients with left-sided breast cancer when the internal mammary nodes were included in the radiation fields because of the steep dose fall off away from the target volume. The average heart volume receiving more than 30 Gy were 2.6 and 16.4% for IMRT and 3D-CRT, respectively (14). The superiority of IMRT over 3D-CRT for left-sided breast cancer was particularly beneficial in patients with a significant heart volume (more than 1 cm) included in the radiation fields. The mean percentage of the heart volume receiving more than 60% of the prescription dose was 2.2 and 4.4% for IMRT and 3D-CRT, respectively (15). The benefit of IMRT to decrease cardiac irradiation while improving target coverage and dose homogeneity was also corroborated in other dosimetric studies (16). As randomized studies demonstrated a significant reduction of side effects during radiotherapy for breast cancer with IMRT compared to 3D-CRT, it is quite possible that long-term cardiac complications may also be reduced in the future with IMRT given its cardiac sparing properties. However, long-term follow-up is needed to confirm this hypothesis.

## Potential Advantages of IGRT for Cardiac Sparing in Patients with Left-Sided Breast Cancer

Image-guided radiotherapy is a tool that can be used in different radiotherapy treatments, including IMRT that gives the possibility of reduction of set up margins, with a better sparing of normal tissue, while promoting dose-escalation to the tumor. Thus, the visualization of the surgical tumor bed as outlined by the fiducial markers or the lumpectomy cavity and the organs at risk (OAR) during radiotherapy may allow the delivery of a higher radiation dose to the areas at risk for recurrence while reducing irradiation of the normal organs such as the heart and lungs. As an illustration, compared to IMRT, IGRT may substantially reduce radiation dose to a small organ such as the cochlea without sacrificing target coverage in patients with head and neck cancer (17). Preliminary results of IGRT for normal organs sparing in patients with breast cancer are encouraging. As the lumpectomy cavity decreases in size during breast irradiation, re-planning for the tumor boost toward the end of whole breast irradiation may decrease the volume of the normal breast irradiated to a higher dose and potentially improve cosmetic results (18). This issue is particularly important in patients who develop a large seroma as a complication of surgery. Visualization of the lumpectomy cavity with daily imaging allows the delivery of a higher radiation dose to the tumor bed while treating the whole breast to a lower dose with the simultaneous integrated boost (SIB) technique. In patients with left-sided breast cancers, the volume of the heart irradiated to a high radiation dose may be significantly reduced. The volume of the heart radiated to 30 Gy (V30) and the mean heart dose were 0.03 and 1.14% and 1.35 and 2.22 Gy for IGRT and 3D-CRT, respectively (19). The cardiac sparing effect of IGRT over 3D-CRT was also corroborated in another study (20). Treatment time may also be shortened with the SIB technique as radiation may be delivered over 28 fractions instead of the conventional 38 fractions (21). Another potential advantage of IGRT over IMRT is its ability to the monitor the patient breathing pattern during radiotherapy with pre-treatment imaging such as cone-beam CT (CBCT). In patients who are able to maintain a deep breath hold in inspiration, the volume of myocardium irradiated may be significantly reduced during radiation because the heart shifts away from the chest wall (22). The feasibility of IGRT for cardiac sparing in patients with left-sided breast cancer was investigated in a prospective study. Nineteen left-sided breast cancer patients were treated with the deep inspiration breath hold (DIBH) technique during IGRT. Compared to the free-breathing (FB) technique, DIBH significantly reduced radiation dose to the heart. The percentage of the left ventricle radiated was 28 and 71% for DIBH and FB, respectively (23). For selected patients with pathological stage T1 infiltrating ductal carcinoma completely resected without nodal involvement who could lay prone during radiotherapy, the tumor cavity and margins can also be treated with IGRT to 30 Gy in five fractions without compromise of loco-regional control (24). As the prone position allows reduction of radiation dose to the heart compared to the supine position (25), IGRT may potentially spare the heart from excessive radiation while shortening the treatment course in patients with left-sided breast cancer (26). The feasibility of IGRT to spare the heart in the prone position with an accelerated partial breast radiotherapy regimen was also corroborated in another study (27). For breast cancer patients with a special chest anatomy such as pectus excavatum or funnel chest where a large heart volume is frequently included in the radiation fields, IGRT may also be beneficial in avoiding excessive cardiac irradiation (28). Thus, daily imaging with IGRT allows delivery of IMRT through various treatment positions and breathing cycles, which may improve cardiac sparing without compromise of the target volume in patients with left-sided breast cancer.

## Clinical Studies of IGRT for Breast Cancer Treatment

Preliminary studies of IGRT for breast cancer treatment have been promising. In a phase II study, 50 patients with stage I–III infiltrating ductal carcinoma of the breast were treated with IGRT in the supine position with hypofractionation. The whole breast and lumpectomy cavity were treated to 40.5 and 48 Gy in 15 fractions with the SIB technique. Daily CBCT was performed to verify the patient set up before each treatment. The constraint for the cardiac dose was D40 less than 3%. In patients with left breast lesions, the dose to the heart was kept to a maximum dose of 27 Gy. Despite a shorter treatment course, only one patient developed a grade 3 skin reaction. There were no complications or loco-regional recurrences after a median follow-up of 12 months (29). The benefit of hypofractionated IGRT for breast cancer was also corroborated in another randomized study (30). In all, 123 stage I and II breast cancer patients were randomized to IGRT (59) and 3D-CRT (64). The patients were treated to 42–51 Gy over 3 weeks and 50–66 Gy over 5–7 weeks with IGRT and 3D-CRT, respectively. Quality of life (QOL) was assessed with questionnaires at 3 months, and 1, 2, and 3 years after treatment. At 3 months post-radiotherapy, patients who had IGRT developed less fatigue and had improved physical and emotional functioning compared to those undergoing 3D-CRT. After a median follow-up of 26 months, the IGRT group still had a better QOL score even though the difference was no longer statistically significant. Thus, despite a shorter treatment course, IGRT may provide a better QOL for breast cancer patients possibly because of improved normal organ sparing. In another randomized study of 59 stage I and II breast cancer patients treated with IGRT (37) and 3D-CRT (32) treated with a similar fractionation, cardiac toxicity was similar for both groups (31). Despite the small number of patients and the short follow-up, these preliminary studies raised intriguing questions about the potential benefit of IGRT to decrease treatment toxicity and to improve QOL in breast cancer patients. It remains to be seen whether the potential of IGRT to reduce cardiac irradiation for left-sided breast cancer may translate into long-term reduction of cardiac complications.

## Identification of Patients Who may Benefit from IGRT for Left-Sided Breast Cancer

Patients with human epidermal growth factor receptor-2 (Her-2) positive breast cancer had improved survival when trastuzumab was added to their chemotherapy regimen. Even though trastuzumab is well tolerated in most patients, a small proportion may develop congestive heart failure and left ventricular dysfunction as complication. As the half-life of trastuzumab is 4 weeks, it will take 16–20 weeks to be cleared from the body and as a result may still be present at clinically significant levels at the time of radiotherapy. Preliminary results suggest that trastuzumab may potentiate the cardiotoxicity of radiation. Even a mean heart dose of 10 Gy may increase the risk of low grade cardiac toxicity when trastuzumab was administered with radiation in patients with left breast cancer (32). In another study of 95 breast cancer patients who had radiotherapy and concurrent trastuzumab, 58 patients experienced left ventricular ejection dysfunction (33). Grade 2 decrease in left ventricular ejection fraction was also observed in 10% of the patients receiving trastuzumab and breast radiotherapy (34). As radiotherapy should not be delayed in Her-2 positive breast cancer patients receiving trastuzumab and the long-term effect of the combined treatment remains unknown, these patients may benefit from IGRT if the tumor is located in the left breast. As the risk cardiac toxicity increases over time following treatment with anthracycline-based chemotherapy and radiotherapy for breast cancer, these patients may also benefit from IGRT to minimize radiation dose to the myocardium (9). However, as most chemotherapy agents have cardiac effects, left-sided breast cancer patients who had a history of chemotherapy and radiotherapy should be monitored closely to determine any deterioration of their cardiac function over time (35). Elderly breast cancer patients (70 years or older) are more likely to have pre-existing cardiovascular morbidities and less likely to receive radiotherapy following surgery compared to younger patients because of the fear of treatment toxicity (36). The elderly breast cancer patients are more likely to benefit from a short course of hypofractionated IGRT to reduce treatment time and to decrease the toxic effect of cardiac irradiation. Such study should be conducted in the future to assess the beneficial effect of IGRT for local control and survival in elderly breast cancer patients.

## Conclusion

Image-guided radiotherapy is a promising new technique of radiation that may significantly decrease cardiac irradiation in patients with left-sided breast cancers and potentially decrease long-term cardiac complications. Future prospective studies should be performed to verify this hypothesis.

## Conflict of Interest Statement

The authors declare that the research was conducted in the absence of any commercial or financial relationships that could be construed as a potential conflict of interest.
